# Supplementary material for: First Identification and Investigation of piRNAs in the Larval Gut of the Asian Honeybee, Apis cerana
Source: Insects. 2022 Dec 23;14(1):16. doi: 10.3390/insects14010016 (PMC9863445; doi:10.3390/insects14010016)
Supplement: Supplementary file 1 [file insects-14-00016-s001.zip › Figure-S1.pdf]

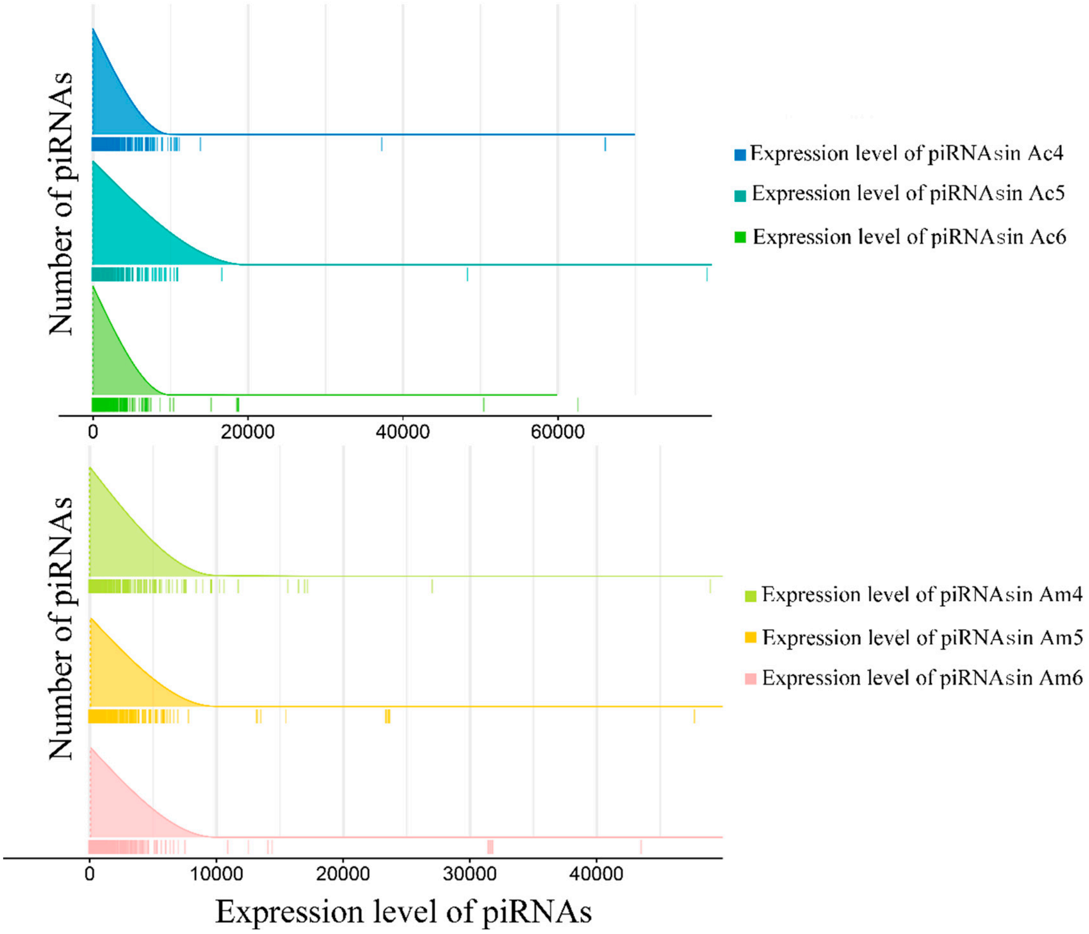

Figure S1. Ridgeline plots of expression levels total piRNAs in *A. c. cerana* and *A. m. ligustica* larval guts. The peak of the ridge indicates the most abundant piRNA in each group, and each line below indicates a piRNA.
